# Insulin-like growth factor binding protein-3 induces senescence by inhibiting telomerase activity in MCF-7 breast cancer cells

**DOI:** 10.1038/s41598-023-35291-5

**Published:** 2023-05-30

**Authors:** Ahreum Kwon, Hyun Wook Chae, Woo Jung Lee, JungHyun Kim, Ye Jin Kim, Jungmin Ahn, Youngman Oh, Ho-Seong Kim

**Affiliations:** 1 Department of Pediatrics, Severance Children’s Hospital, Endocrine Research Institute, College of Medicine, Yonsei University, Seoul, 03722 South Korea; 2 Department of Pathology, School of Medicine, Virginia Commonwealth University, Richmond, VA 23298 USA; 3 Department of Pediatrics, Endocrine Research Institute, College of Medicine, Yonsei University, 50-1 Yonsei-ro, Seodaemun-gu, Seoul, 03722 South Korea

**Keywords:** Cancer, Cell biology

## Abstract

Insulin-like growth factor binding protein-3 (IGFBP-3) has been known to inhibit cell proliferation and exert tumor-suppressing effects depending on the cell type. In this study, we aimed to show that IGFBP-3 induces cellular senescence via suppression of the telomerase activity, thereby inhibiting MCF-7 breast cancer cell proliferation. We found that the induction of IGFBP-3 in MCF-7 cells enhanced the loss of cell viability. Flow cytometry revealed a higher percentage of non-cycling cells among IGFBP-3-expressing cells than among controls. IGFBP-3 induction also resulted in morphological alterations, such as a flattened cytoplasm and increased granularity, suggesting that IGFBP-3 induces a senescence-like phenotype. The percentage of IGFBP-3 expressing cells with senescence-associated β-galactosidase activity was 3.4 times higher than control cells. Telomeric repeat amplification and real-time PCR showed that IGFBP-3 decreased telomerase activity by reducing the levels of the RNA component (hTR) and catalytic protein component with reverse transcriptase activity (hTERT) of telomerase in a dose-dependent manner. These results suggest that IGFBP-3 is a negative regulator of MCF-7 breast cancer cell growth by inducing senescence through telomerase suppression.

## Introduction

Insulin-like growth factor binding protein (IGFBP-3) is a multifunctional protein involved in critical cellular processes, including proliferation, differentiation, survival, migration, senescence, autophagy, and angiogenesis^1–3^. IGFBP-3 is known to modulate the actions of insulin-like growth factors (IGFs) by regulating their availability to the IGF-I receptors^4,5^. Additionally, IGFBP-3 interacts with several non-IGF proteins and growth factors to exert IGF-independent actions^1–3^. IGFBP-3 has been reported to exert tumor-suppressing or promoting effects depending on the cell type, cellular environment, and status of coupling with other cell signaling systems^3^. However, IGFBP-3 primarily functions as a tumor suppressor, as supported by the findings that IGFBP-3 alterations at transcriptional and post-translational stages have implications in the pathogenesis of several malignancies, including breast, prostate, and lung cancers^6^. Increasing evidence suggests that IGFBP-3 interacts with a range of proteins or signaling cascades involved in apoptosis and cell cycle regulation^7–12^. IGFBP-3 has been reported to inhibit cell proliferation through apoptosis induction by increasing the ratio of pro-apoptotic to anti-apoptotic proteins^13^ or activating caspases^14^. Furthermore, IGFBP-3 may inhibit cell proliferation by modulating the cell cycle. IGFBP-3 has been shown to induce cell cycle arrest by decreasing p34cdc2, a protein critical for cell cycle arrest^15^; increasing the cyclin-dependent kinase (CDK) inhibitory protein, p21/WAF1^16^; and decreasing cell cycle-regulating proteins, including cyclin D1, cyclin D3, cyclin E, cyclin A, cyclin-dependent kinase (CDK) 2, CDK4, and total and phospho-pRb^17^. The observed anti-proliferative and pro-apoptotic functions of IGFBP-3 may provide insight into its significance in the pathophysiology and etiology of various human diseases, including cancer, diabetes, vascular disease, asthma, and growth disorders^18–20^. However, the mechanism for the growth inhibitory action of IGFBP-3 in various cell systems remains unclear.

Previous research has linked IGFBP-3 to cellular senescence in various cell types, including fibroblasts^21^, epithelial cells^22^, and endothelial cells^23,24^. The IGFBP-3 enhancer element, a transcriptional regulatory element identified in the 5′ untranslated region of the IGFBP-3 gene, has been shown to activate IGFBP-3 expression in senescent fibroblasts^25^. Moreover, ectopic expression of IGFBP-3 in young human umbilical vein endothelial cells induces senescence, suggesting that IGFBP-3 may be a rate-limiting regulator of endothelial cell senescence^24^. Using a secretome proteomics approach, IGFBP-3 has also been identified as a secreted mediator of breast cancer cell senescence following chemotherapeutic drug treatment^26^.

Senescence is promoted by a shortening of telomere length and decreased telomerase activity. Telomerase activity is the primary mechanism for telomere maintenance in eukaryotes^27^. Telomere length shortens during differentiation because of the downregulation of telomerase activity in somatic cells but not germline cells. As a result, following each replication, proliferating cells lose approximately 50–200 bp of telomeric DNA. In culture, telomerase-negative normal human cells undergo replicative senescence, a telomere length-dependent growth arrest. In addition, diverse stressful stimuli can induce a senescence-like response known as stress-induced premature senescence. Therefore, senescence is thought to function as a protective mechanism against the accumulation of damaged DNA and transformation of cells, in addition to being an underlying cause of aging^28^. Telomerase activity can be detected in early human development, such as in fetal tissue cells, stem cells, and some types of germ cells and epithelial cells, although it is downregulated in most human adult tissues^29,30^. In contrast, telomerase activity has been reported in most human tumor cell lines^31,32^. Therefore, reduced telomerase activity leads to normal cell senescence, whereas increased telomerase activity promotes tumor cell proliferation.

This study demonstrates that IGFBP-3 inhibits cell proliferation through senescence in MCF-7 human breast cancer cells by inhibiting telomerase activity. Furthermore, we elucidate the potential mechanism by which IGFBP-3 alters the expression of telomerase components, such as human telomerase RNA (hTR) and human telomerase reverse transcriptase (hTERT).

## Results

### IGFBP-3 reduces MCF-7 cell viability

**Figure 1:**
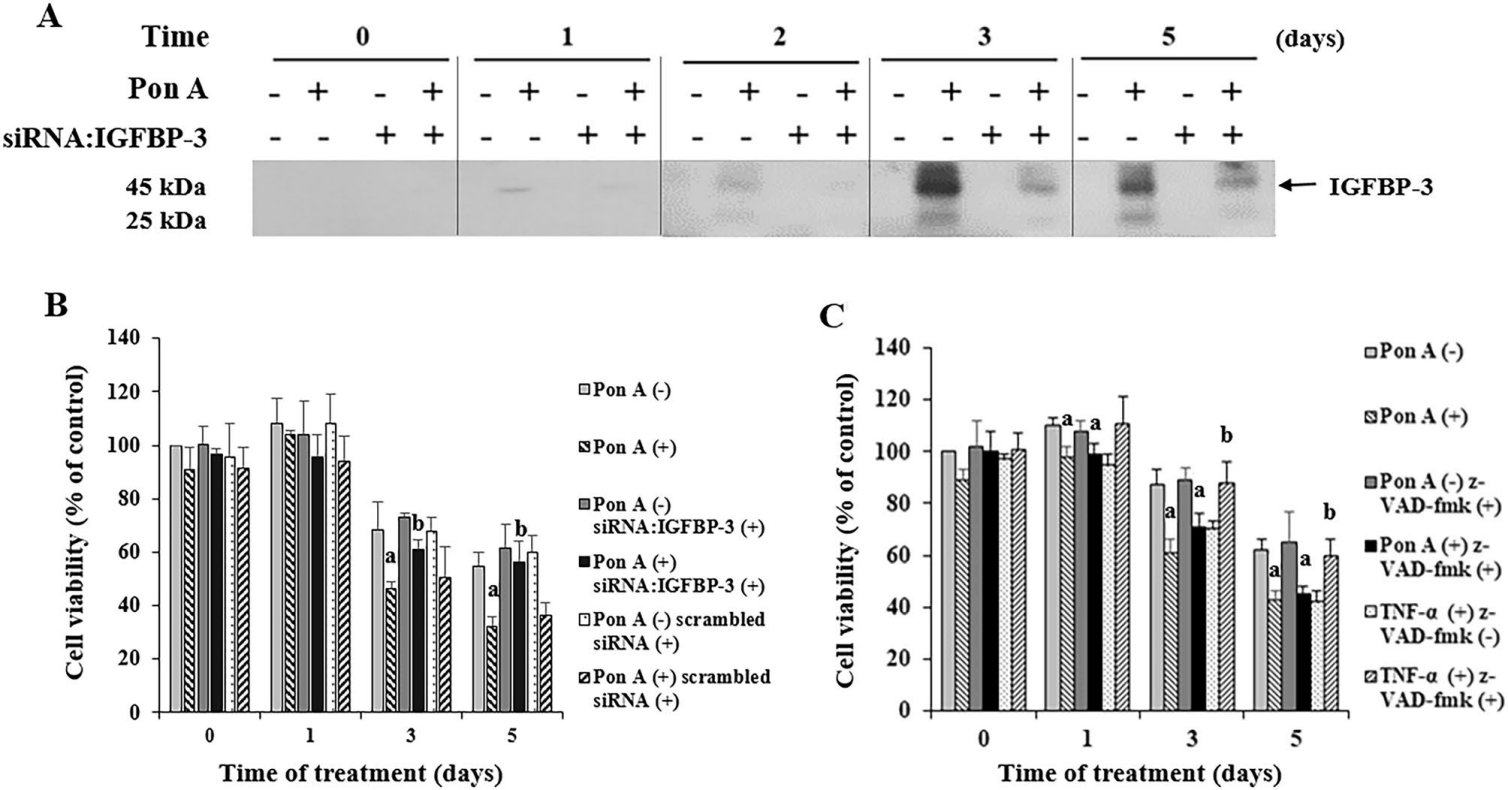
IGFBP-3 enhances the loss of MCF-7 cell viability by a mechanism other than caspase-mediated apoptosis. (**A**) IGFBP-3 expression in MCF-7 cells following the addition of ponasterone A with or without siRNA:IGFBP-3 at the indicated times. Confluent MCF-7 cells were transfected with 50 nM siRNA:IGFBP-3 and cultured in DMEM supplemented with 10% FBS. After 24 h, the cells were incubated with phenol red-free SFM in the presence or absence of 15 µM ponasterone A for the indicated times. Two western blots were combined to display the levels of 20 samples in one figure. (**B**) IGFBP-3 enhances the loss of viability in stably transfected MCF-7 cells. Cells were incubated with phenol red-free SFM after adding 15 µM ponasterone A with or without 50 nM siRNA:IGFBP-3, or scrambled siRNA for the indicated times before the cell viability assay. On days 3 and 5, IGFBP-3-expressing cells had significantly lower cell growth than control cells. The inhibitory effect of IGFBP-3 was abolished by siRNA:IGFBP-3 (p < 0.05), but not by scrambled siRNA. A. p < 0.05, vs. ponasterone A-untreated cells; b. p < 0.05, vs. ponasterone A-treated cells. (**C**) IGFBP-3 partially inhibits cell viability independent of caspase-mediated apoptosis. Cells were incubated with phenol red-free SFM after the addition of 15 µM ponasterone A or TNF-α (30 ng/mL) with or without 25 µM z-VAD-fmk for the indicated times. The inhibitory effect of IGFBP-3 was partially abolished by co-treatment with z-VAD-fmk, whereas the inhibitory effect of TNF-α was abolished by z-VAD-fmk (p < 0.05). a. p < 0.05, vs. ponasterone A-untreated cells; b. p < 0.05, vs. TNF-α-treated cells without z-VAD-fmk. Multiple comparisons were performed using one-way ANOVA with Bonferroni post-hoc analysis. Each bar represents the mean ± SD of three independent experiments.

We added ponasterone A to an MCF-7-derived cell line to express endogenous IGFBP-3 in an inducible manner. IGFBP-3 concentrations in the culture medium were increased on days 1, 2, 3, and 5 with the addition of ponasterone A (Fig. 1A, Supplemetary Fig. S1, Table S1). Co-treatment with ponasterone A and siRNA: IGFBP-3 prevented the increase of IGFBP-3 expression at all the indicated times (Fig. 1A, Supplementary Fig. S1, Table S1). Ponasterone A-induced IGFBP-3 expression resulted in a significant reduction of cell viability, ranging from 41 to 49% on days 3 and 5, when compared to cells without ponasterone A (p < 0.05) (Fig. 1B). This reduction of cell viability was abolished by co-treatment with siRNA:IGFBP-3, but not scrambled siRNA, demonstrating the specific effect of IGFBP-3 on the loss of cell viability (Fig. 1B).

### Effect of IGFBP-3 on cell viability is mediated by a mechanism other than apoptosis

Previous studies have reported that IGFBP-3 induces apoptosis in various cell systems^10–14^. Therefore, to determine whether only apoptosis contributed to the loss of cell viability by IGFBP-3, we performed cell viability assays using the pan-caspase inhibitor, z-VAD-fmk. The expression of IGFBP-3 or the addition of TNF-α (30 ng/mL), which is known to activate the caspase system, significantly reduced cell viability compared to control cells (72.8% and 82.0% on day 3, p < 0.01 and p < 0.05, respectively) (Fig. 1C). However, treatment with z-VAD-fmk completely abolished the inhibitory effect of TNF-α (p < 0.05) but partially abolished the inhibitory effect of IGFBP-3, suggesting that IGFBP-3 decreased cell viability by a mechanism other than caspase-mediated apoptosis.

### IGFBP-3 increases the fraction of non-cycling cells

**Figure 2:**
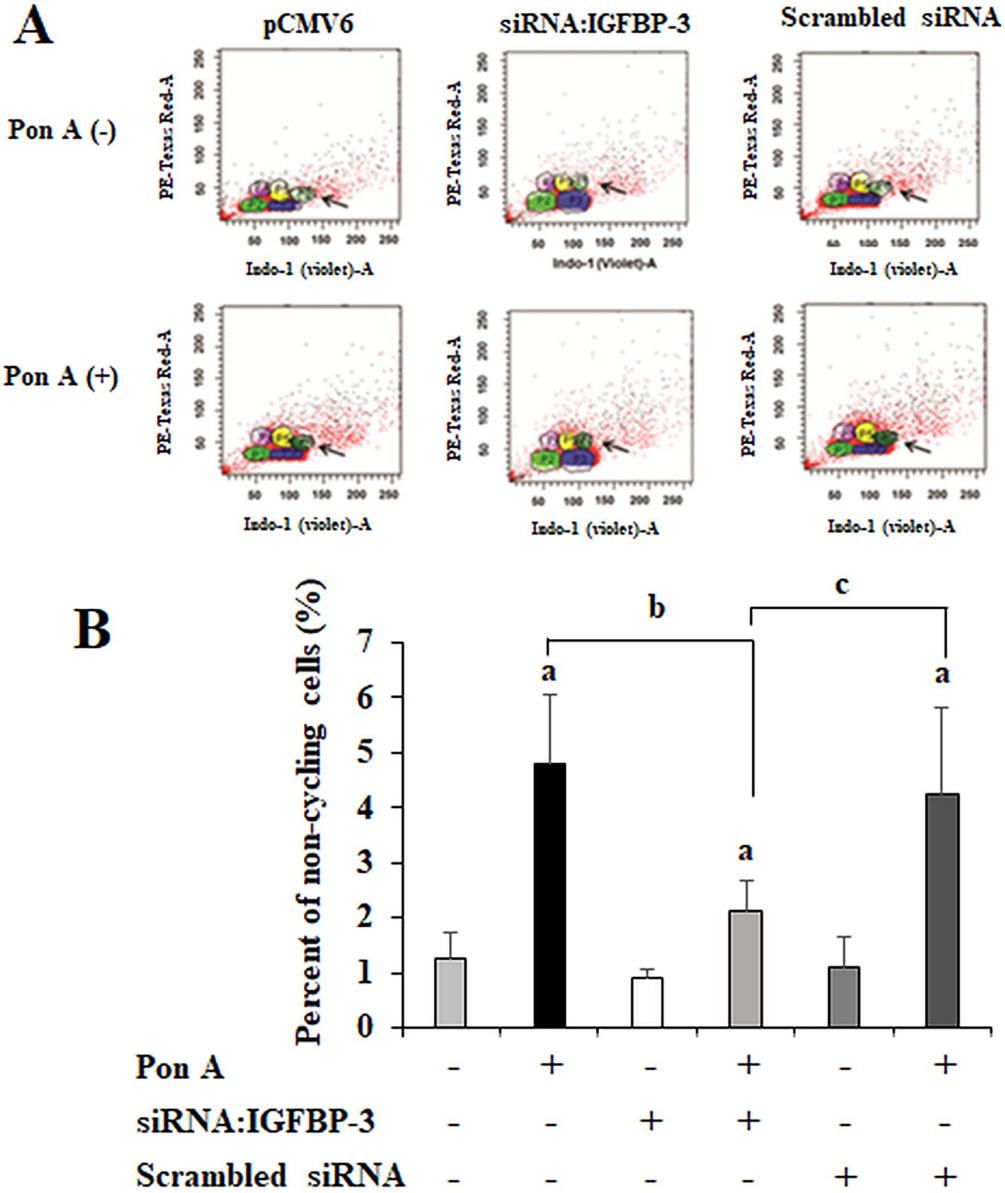
IGFBP-3 increases the fraction of non-cycling cells. (**A**) The percentage of non-cycling cells was determined using the BrdU–Hoechst quenching technique in the presence or absence of siRNA:IGFBP-3. (**B**) The percentage of non-cycling cells increased from 1.25% in the absence of ponasterone A to 4.8% in its presence (p < 0.005). The percentage of non-cycling cells in the culture was reduced from 4.8 to 2.13% by co-treatment with siRNA:IGFBP-3 (p < 0.01), but not by scrambled siRNA. a. p < 0.05, ponasterone A-untreated cells vs. ponasterone A-treated cells; b. p < 0.05, ponasterone A-treated cells vs. ponasterone A-treated cells with siRNA:IGFBP-3; c. p < 0.05, ponasterone A-treated cells with scrambled siRNA vs. ponasterone A-treated cells with siRNA:IGFBP-3. Multiple comparisons were performed using one-way ANOVA with Bonferroni post-hoc analysis. Each bar represents the mean ± SD of three independent experiments.

Our previous study demonstrated that IGFBP-3 inhibits MCF-7 cell proliferation by inducing G1 cell cycle arrest^17^. Next, to determine whether IGFBP-3 induces senescence, a permanent exit from the cell cycle, we assessed cells ectopically expressing IGFBP-3 for an increase in the frequency of non-cycling cells using flow cytometry (Fig. 2A). The percentage of non-cycling cells increased from 1.25% (in the absence of ponasterone A) to 4.8% (in the presence of ponasterone A) (p < 0.01) (Fig. 2B). The percentage of non-cycling cells induced by IGFBP-3 was reduced from 4.8% to 2.13% by co-treatment with siRNA:IGFBP-3, but not scrambled siRNA, suggesting that this effect was specifically owing to increased IGFBP-3 expression. These results indicate that the induced expression of IGFBP-3 increases the fraction of non-cycling cells in this cell system, suggesting that IGFBP-3 may induce senescence in some cells.

### IGFBP-3 induces a senescence-like phenotype

**Figure 3:**
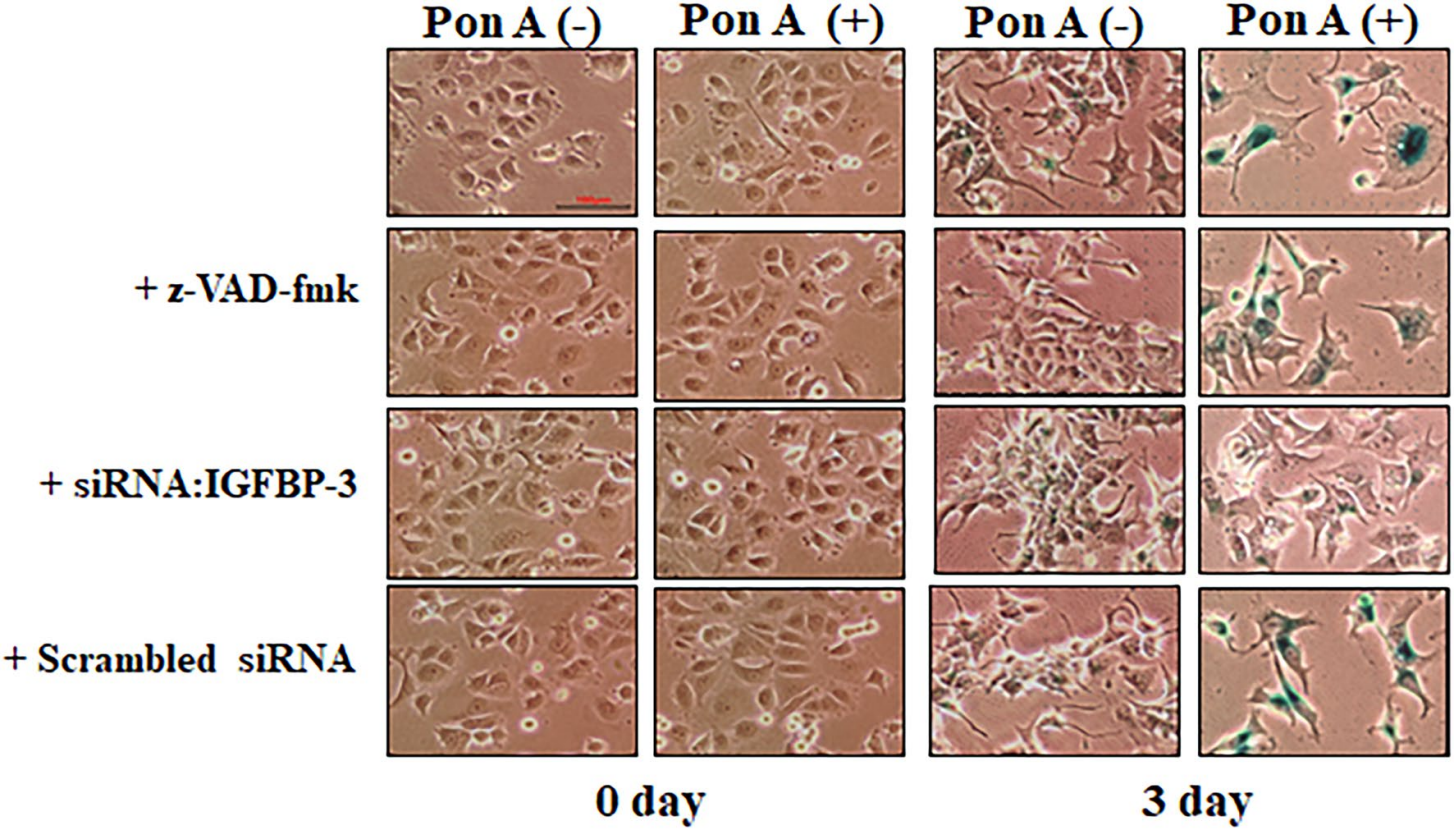
IGFBP-3 induces a senescence-like phenotype by a mechanism independent of caspase-mediated apoptosis. Morphological alterations were observed in IGFBP-3-induced cells in the presence or absence of pan-caspase inhibitor, siRNA:IGFBP-3, or scrambled siRNA. Induction of IGFBP-3 resulted in morphological changes, such as a flattened cytoplasm and increased granularity similar to that observed in senescent cells on day 3. The senescence-like phenotype was abolished by co-treatment with siRNA:IGFBP-3, whereas a pan-caspase inhibitor or scrambled siRNA did not abolish these effects. Cells exhibiting blue color in the slides had senescence-associated ß-galactosidase staining. Original magnification, × 200.

IGFBP-3 induction resulted in morphological alterations, such as flattened cytoplasm and increased granularity (Fig. 3), comparable to those reported in senescent cells^33^. These findings imply that IGFBP-3 expression induces a senescence-like phenotype. These morphological changes were abolished by siRNA:IGFBP-3, but not z-VAD-fmk or scrambled siRNA, suggesting that the induction of senescence-like phenotype by IGFBP-3 is specific, and apoptosis is not the only mechanism responsible for reduced proliferation in cells ectopically expressing IGFBP-3.

### IGFBP-3 increases senescence-associated ß-galactosidase activity

**Figure 4:**
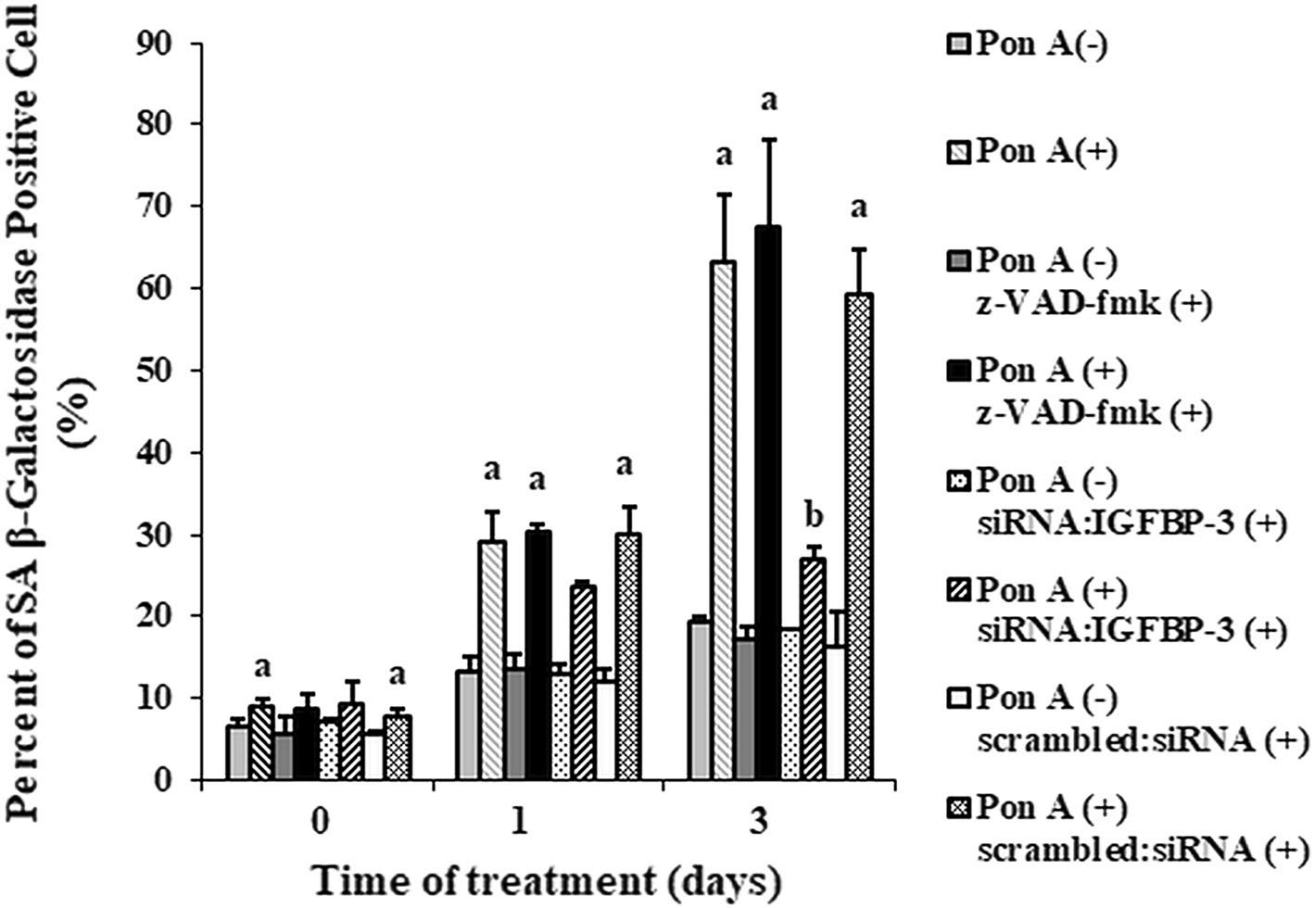
IGFBP-3 increases senescence-associated (SA)-ß-galactosidase-positive cells, a relatively specific marker of senescence. The percentages of IGFBP-3-expressing cells with SA-β-galactosidase activity were determined in the presence or absence of pan-caspase inhibitor, siRNA:IGFBP-3, or scrambled siRNA. The percentage of ponasterone A-treated cells with senescence-associated β-galactosidase activity was 3.4 times higher than control cells on day 3. The effect of IGFBP-3 on SA-β-galactosidase activity was abolished by co-treatment with siRNA:IGFBP-3, but not with pan-caspase inhibitor or scrambled siRNA (p < 0.01, ponasterone A-treated cells on day 3 vs. ponasterone A + siRNA:IGFBP-3 treated cells on day 3). a. p < 0.05, vs. ponasterone A-untreated cells; b. p < 0.05, vs. ponasterone A-treated cells. Multiple comparisons were performed using one-way ANOVA with Bonferroni post-hoc analysis. Each bar represents the mean ± SD of three independent experiments.

We determined the percentage of cells containing SA-ß-galactosidase, a marker of senescence, using immunocytochemistry^34^. On day 3, the percentage of IGFBP-3-expressing cells with SA-ß-galactosidase activity was 3.4 times higher than that of control cells (p < 0.01) (Fig. 4). Increased SA-ß-galactosidase activity by IGFBP-3 was abolished by the addition of siRNA:IGFBP-3 by about 43% on day 3 (p < 0.01), whereas treatment with scrambled siRNA had no effect. These results suggest that IGFBP-3 induces senescence.

### IGFBP-3 decreases telomerase activity

**Figure 5:**
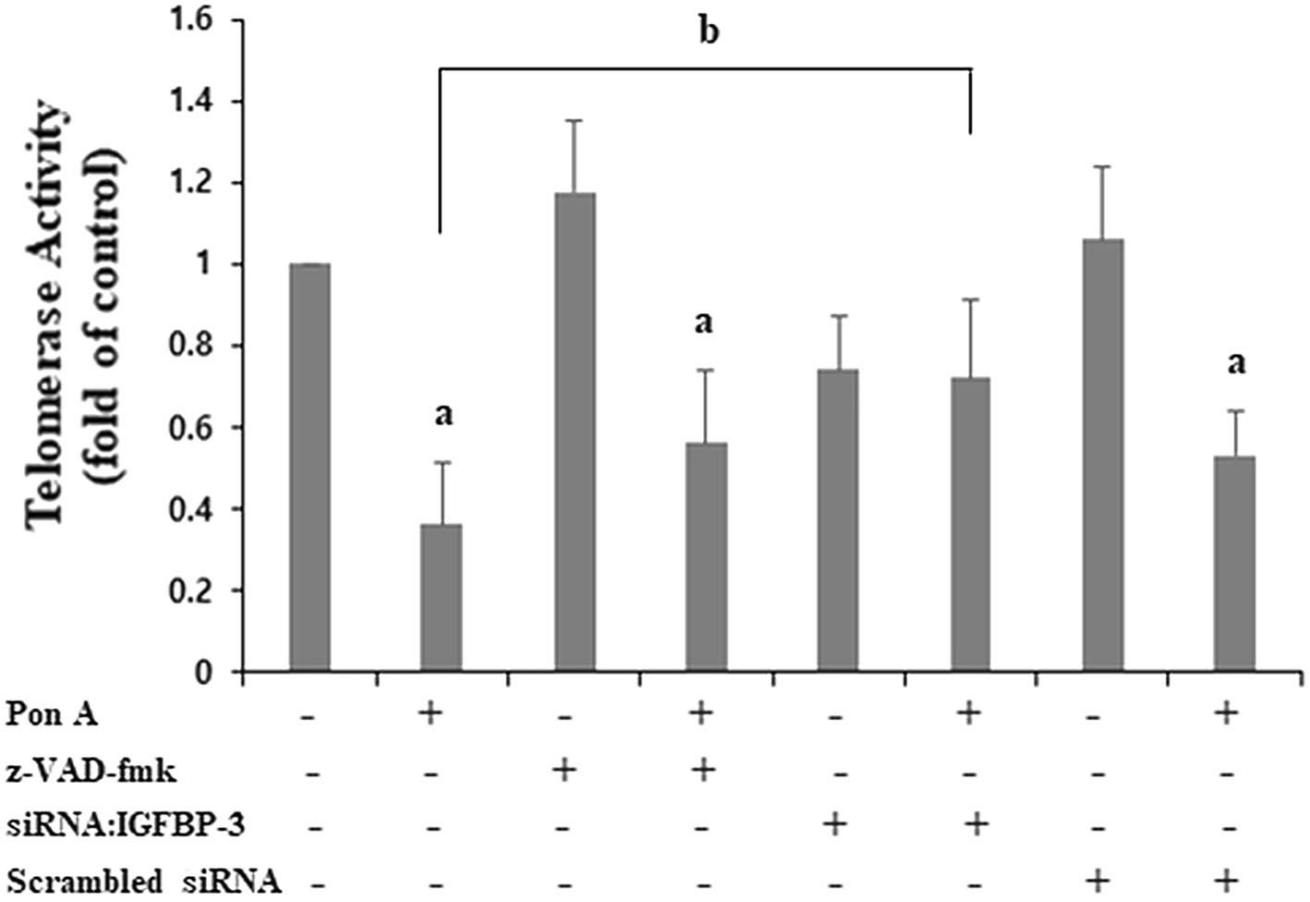
IGFBP-3 decreases telomerase activity independent of caspase-mediated apoptosis. Relative quantification of telomerase activity levels after the induction of IGFBP-3 in the presence or absence of pan-caspase inhibitor (z-VAD-fmk), siRNA:IGFBP-3, or scrambled siRNA. Induction of IGFBP-3 decreased telomerase activity. The effect of IGFBP-3 on telomerase activity was prevented by co-treatment with siRNA:IGFBP-3, but not abolished by the pan-caspase inhibitor or scrambled siRNA. a. p < 0.05, vs. ponasterone A-untreated cells; b. p < 0.05, vs. ponasterone A-treated cells. Multiple comparisons were performed using one-way ANOVA with Bonferroni post-hoc analysis. Each bar represents the mean ± SD of three independent experiments.

Next, we used a TRAP Photometric Enzyme Immunoassay to determine whether IGFBP-3 induced senescence by modulating telomerase activity. The induction of IGFBP-3 significantly decreased telomerase activity compared to cells without ponasterone A (p < 0.01) (Fig. 5), suggesting that decreased telomerase activity in IGFBP-3-induced MCF-7 cells may be one of the mechanisms inducing senescence. The decrease in telomerase activity by IGFBP-3 was abolished by co-treatment with siRNA:IGFBP-3, but not z-VAD-fmk or scrambled siRNA. These results suggest that the modulation of telomerase activity by IGFBP-3 is specific and independent of caspase-mediated apoptosis.

### IGFBP-3 decreases reverse transcriptase and RNA components of telomerase in IGFBP-3 transfected MCF-7 cells

**Figure 6:**
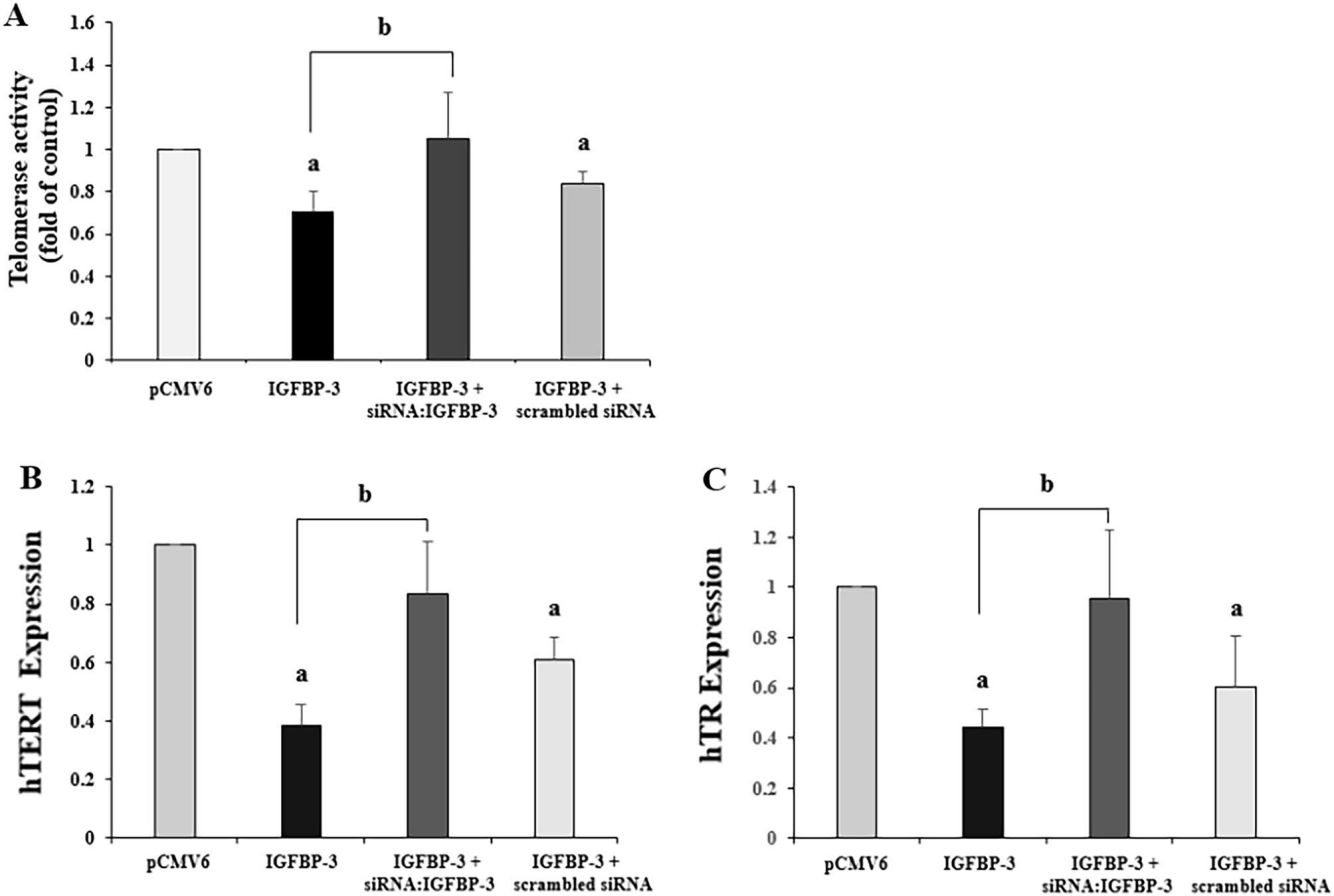
IGFBP-3 decreases telomerase activity and alters human telomerase reverse transcriptase (hTERT) and human telomerase RNA (hTR) levels. IGFBP-3 plasmid was transfected to MCF-7 cells. Telomerase activity decreased in IGFBP-3 plasmid-transfected MCF-7 cells (**A**). In addition, IGFBP-3 decreased hTERT (**B**) and hTR (**C**) mRNA levels, as revealed by reverse-transcription quantitative PCR. The effect of IGFBP-3 on hTERT and hTR RNA levels was prevented by co-treatment with siRNA:IGFBP-3 but not scrambled siRNA, suggesting that IGFBP-3 has specific effects. a. p < 0.05, vs. empty-vector-transfected cells; b. p < 0.05, vs. IGFBP-3 transfected cells. Multiple comparisons were performed using one-way ANOVA with Bonferroni post-hoc analysis. Each bar represents the mean ± SD of three independent experiments.

We determined the levels of reverse transcriptase (hTERT) and RNA (hTR) to elucidate the potential mechanism by which IGFBP-3 affects telomerase activity. Telomerase activity decreased in IGFBP-3-transfected MCF-7 cells. However, the addition of siRNA:IGFBP-3 to IGFBP-3 transfected MCF-7 cells did not affect the telomerase activity of the cells (Fig. 6A). In reverse-transcription quantitative PCR, IGFBP-3-transfected MCF-7 cells decreased the mRNA levels of hTERT and hTR (Fig. 6B,C). The addition of siRNA:IGFBP-3 to the IGFBP-3-transfected cells did not decrease the mRNA levels of hTERT and hTR, whereas the addition of scrambled siRNA decreased these mRNA levels (Fig. 6B,C). These results suggest that IGFBP-3 reduces telomerase activity and alters the level of telomerase components, including reverse transcriptase and RNA.

### Increased telomerase activity by overexpression of hTERT in normal breast cells is inhibited by exogenous IGFBP-3, thereby increasing senescence in a dose-dependent manner

**Figure 7:**
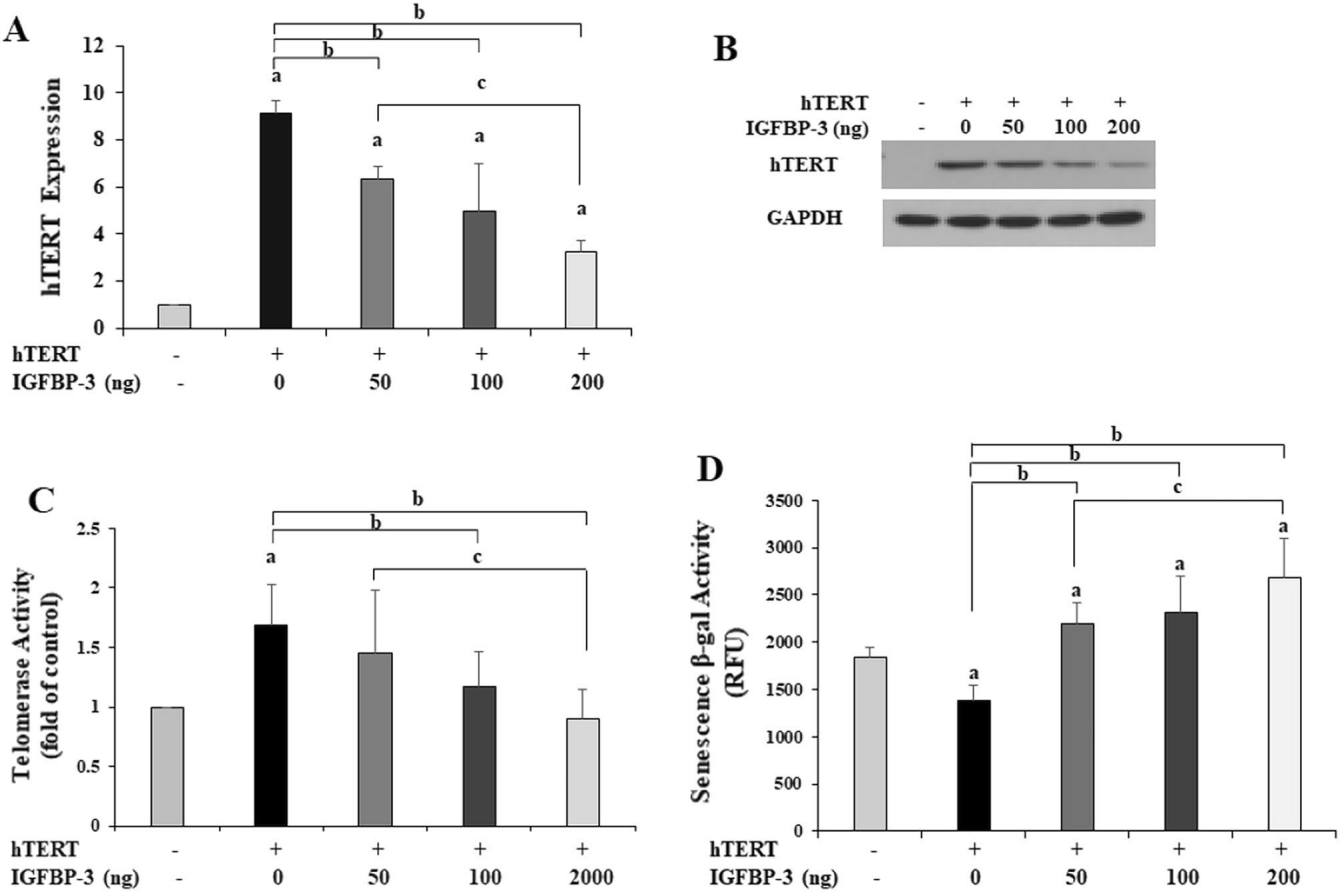
The hTERT plasmid was transfected into human normal breast cells (MCF-12F) to induce hTERT overexpression. Subsequently, various concentrations of exogenous IGFBP-3 were added. Exogenous IGFBP-3 decreased the levels of hTERT mRNA (**A**) and protein (**B**) in a dose-dependent manner. In hTERT overexpressed cells, IGFBP-3 decreased telomerase activity (**C**) and increased senescence-associated ß-galactosidase activity (**D**) in a dose-dependent manner. a. p < 0.05, vs. normal cells; b. p < 0.05, vs. hTERT overexpressed cells without IGFBP-3; c. p < 0.05, vs. hTERT overexpressed cells with 50 ng IGFBP-3. Multiple comparisons were performed using one-way ANOVA with Bonferroni post-hoc analysis. Each bar represents the mean ± SD of three independent experiments.

Next, we transfected hTERT into MCF-12F human normal breast cells to overexpress hTERT. We determined the mRNA and protein levels of hTERT and telomerase activity and SA β-galactosidase activity after the addition of exogenous IGFBP-3 at various concentrations. Exogenous IGFBP-3 decreased the levels of hTERT mRNA (Fig. 7A) and protein (Fig. 7B, Supplementary Fig. S2) in a dose-dependent manner. Along with this, telomerase activity was decreased and SA-ß-galactosidase activity was increased in a IGFBP-3 dose-dependent manner (Fig. 7C,D). These results suggest that IGFBP-3 induces cellular senescence through reducing telomerase activity in a dose-dependent manner.

## Discussion

The present study revealed that IGFBP-3 decreases cell viability of the MCF-7 human breast cancer cell line via inducing senescence. IGFBP-3 expression resulted in morphological changes typical of senescent cells, such as flattened cytoplasm and increased granularity, as well as a marked increase in the percentage of cells expressing SA-ß-galactosidase. Furthermore, BrdU–Hoechst flow cytometry showed that IGFBP-3 increased the fraction of non-cycling cells, implying that IGFBP-3 induces senescence. Previous research suggests that IGFBP-3 inhibits cell proliferation by inducing apoptosis^13,14^. In the present study, we used a pan-caspase inhibitor (zVAD-fmk) to demonstrate that the inhibitory effect of IGFBP-3 on cell viability could be attributable to another mechanism in addition to caspase-mediated apoptosis. Treatment with a pan-caspase inhibitor (zVAD-fmk) only partially abolished the loss of cell viability effect of IGFBP-3, whereas it abolished the growth-inhibitory effect of TNF-α. These results suggest that IGFBP-3 enhances the loss of cell viability partly by inducing senescence, in addition to inducing caspase-mediated apoptosis in MCF-7 human breast cancer cells, as previously reported^13,14^.

Cellular senescence is the phenomenon of diploid cells undergoing a permanent cell cycle arrest that limits their capacity to proliferate^35^. Senescent cells show no response to mitogens and are permanently arrested in the G0/G1 phase of the cell cycle. This permanent growth arrest, resulting from telomeric shortening in each cell division, is referred to as replicative senescence^27,28^. In addition, stress-induced premature or accelerated senescence can be observed in cells exposed to oncogenic mutations, oxidative stress, suboptimal culture conditions, or DNA damaging agents^35,36^. Although stress-induced premature senescence was assumed to be independent of telomere length, the fact that stressors can either accelerate telomere shortening^37^ or uncap telomeres^38^ suggests that stress-induced premature senescence can also be telomere-dependent, similar to replicative senescence. Recently, the concept of senescence has been extended to post-mitotic cells beyond cell cycle arrest^39^, as most cells require the induction of both the senescence-associated secretory phenotype (SASP)^40^ and senescence-associated mitochondrial dysfunction^41^ to maintain a prolonged cell cycle arrest. Although SASP facilitates the maintenance and spread of senescence, promoting tissue remodeling and repair^42,43^, it also contributes to tissue dysfunction, age-related tissue degeneration, and tumorigenesis^44^. Furthermore, epigenetic reprogramming and chromatin remodeling occur during senescence and stabilize other senescence processes by promoting mitochondrial dysfunction and SASP production^45,46^. Therefore, cellular senescence is defined as a cellular response to cellular stresses and damage^47^ to function as an additional barrier against the accumulation of damaged DNA, resulting in the transformation of cells, and an underlying cause of aging^28^.

IGFBP-3 may be involved in both replicative senescence and stress-induced premature senescence. Previous studies have suggested that IGFBP-3 expression increases with age in human dermal fibroblasts, epithelial cells, and umbilical vein endothelial cells^22–24^, as well as in breast cancer cells treated with chemotherapeutic drugs^26^ and premature senescent fibroblasts exposed to tert-butylhydroperoxide and ethanol^48^. Furthermore, despite the complexity and diversity of senescence, general evidence suggests that p53 and/or pRb proteins, along with their regulators, such as p21, p16, and ADP ribosylation factor (ARF), are involved in senescence progression^36,37^. Our previous study demonstrated that IGFBP-3 induced G1 cell cycle arrest by inhibiting cyclin D1, cyclin D3, cyclin E, cyclin A, CDK2, CDK4, total and phospho-pRb, and increasing p21 and p16 in MCF-7 breast cancer cells^17^. This study suggests that pRb pathways, at least in part, may be involved in IGFBP-3-induced senescence, as the G1 cell cycle arrest includes senescence responses. The specific molecular mechanism of IGFBP-3-induced senescence remains to be established.

Senescence is linked to telomere length and dysfunction. Telomere shortening with each replication cycle results from the inability of DNA polymerase, telomere dysfunction by either a change of telomeric DNA structure or sequence, or depleted and mutated telomere proteins, leading to permanent growth arrest. These mechanisms may reduce the risk of tumor formation^36^. Telomerase, a ribonucleoprotein complex with reverse transcriptase activity, can catalyze the synthesis of telomeric DNA onto the ends of chromosomes, providing unlimited proliferative capacity to the cells to avoid senescence^27,28^. In most normal human somatic cells, telomerase activity is diminished^49^, and the physiological shortening of telomeres in normal cells triggers replicative senescence or cell death^50^. In contrast, in most human cancer cells, the relatively high level of telomerase activity sustains the proliferation of the cancer cells^32^. In addition, a feed-forward transcriptional loop exists between oncogenes and TERT in cancer cells, which is functionally critical for oncogenesis^51^. In the present study, we aimed to determine whether IGFBP-3 modulates telomerase activity in MCF-7 human breast cancer cells during senescence induction. We demonstrated that the expression of IGFBP-3 decreased telomerase activity using TRAP assays. The present study further demonstrated that IGFBP-3 inhibits telomerase activity by modulating the expression of telomerase components, such as hTR and hTERT, at the mRNA and protein levels. In addition, we found that exogenous IGFBP-3 decreases hTERT mRNA and protein levels and telomerase activity in hTERT-overexpressed normal breast cells. Direct telomere shortening by IGFBP-3 was not confirmed in this study. However, reduced TERT has been reported to prevent cell proliferation without affecting telomere length^32^. Therefore, our results suggest that IGFBP-3-induced inhibition of telomerase activity is a potential mechanism for IGFBP-3-induced senescence in MCF-7 cells. Furthermore, the mechanism underlying the potential inhibition of telomerase by IGFBP-3 in MCF-7 cells remains elusive. Additionally, post-translational modifications (such as phosphorylation, glycosylation, and acetylation) of IGFBP-3 may influence protein activity, interaction, and localization, thereby functioning as a key regulator for IGFBP-3 action^52^. This study did not investigate the occurrence and identification of post-translational modifications; therefore, the biological consequences of post-translational modifications on IGFBP-3 should be elucidated in further studies.

Notably, inverse correlation results suggested that TERT overexpression upregulated several IGF signaling proteins including IGFBP-3 in skeletal stem cells, whereas telomerase deficiency led to a reduction in IGF signaling proteins in osteoblastic cells^53^. This result suggests that impairment of IGF signaling owing to reduced telomerase activity is one of the causes of age-related reduced osteoblast function and bone formation. This is considered an example of organ aging owing to cellular senescence caused by the physiological shortening of telomeres in normal cells, as mentioned earlier in normal human somatic cells. Importantly, the association and causality between IGFBP-3 and telomerase activity may vary depending on the cell type. Therefore, taking this into consideration, future studies on IGF signaling and telomerase activity, including IGFBP-3, should be conducted and analyzed in various ways. Additionally, it is necessary to investigate whether IGF is involved in the mechanism of reducing telomerase activity by IGFBP-3 in MCF-7 cells, which is a result of this study.

This study demonstrated that IGFBP-3 induces senescence in MCF-7 human breast cancer cells by inhibiting telomerase activity, thereby reducing cell viability. In previous studies, IGFBP-3 has been reported to inhibit cell proliferation through the induction of apoptosis or cell cycle arrest. The demonstration of the involvement of IGFBP-3 in the senescence of breast cancer cells in this study presents further evidence of a potential mechanism for the loss of cell viability effect of IGFBP-3, and a potential therapeutic strategy for cancer treatment.

## Materials and methods

### Reagents and antibodies

Dulbecco’s modified Eagle medium (DMEM) and trypsin–EDTA (0.5% trypsin, 5.3 mM EDTA) were purchased from Invitrogen (Carlsbad, CA, USA). DMEM:F-12 media was purchased from Thermo Fisher Scientific (Waltham, MA, USA). Tissue culture reagents and plastics were obtained from Mediatech (Herndon, VA, USA), Becton Dickinson (Franklin Lakes, NJ, USA), Nunc (Naperville, IL, USA), and Sigma-Aldrich (Saint Louis, MO, USA). The ecdysone-inducible expression system was purchased from Invitrogen. CellTiter 96 cell proliferation assay was purchased from Promega (Madison, WI, USA) and z-VAD-fluoromethylketone (fmk) (broad-spectrum caspase inhibitor) from BIOMOL Research Laboratories (Plymouth Meeting, PA, USA). A human IGFBP-3 antibody was purchased from Abcam (Cambridge, MA, USA). A human TERT antibody and Horseradish peroxidase-labeled IgG were purchased from Santa Cruz Biotechnology (Santa Cruz, CA, USA). IGFBP-3 recombinant protein was purchased from Eagle Biosciences (Amherst, NH, USA).

### Cell culture

The MCF-7 human breast cancer cell line was obtained from American Type Culture Collection (ATCC; Rockville, MA, USA). The MCF-7 cell, an estrogen receptor-positive cell line, shows little-to-no secretion of IGFBP-3 under basal condition^54,55^. The cells were maintained in DMEM supplemented with 4.5 g/L glucose, 110 mg/L sodium pyruvate, and 10% fetal bovine serum (FBS). The cells were cultured at 37 °C in 5% CO_2_ and 100% humidity. At 60–70% confluence, the cells were washed with fresh serum-free media (SFM) and then incubated in phenol red-free SFM in the presence of 15 µM ponasterone A for an additional 72 h to express IGFBP-3.

### Establishment of an MCF-7-derived cell line inducibly expressing IGFBP-3

An MCF-7-derived cell line inducibly expressing IGFBP-3 was established as previously described^14^. A cDNA fragment containing a 1.5-kb human IGFBP-3 (equivalent to nucleotides 674–2191 of the cDNA sequence) was ligated into the pIND expression vector containing five modified ecdysone response elements. This construct was co-transfected with a second plasmid (pVgRXR), expressing the ecdysone receptor (VgEcR) and the retinoid X receptor (RXR) into MCF-7 cells using the FuGENE 6 transfection reagent (Roche, Indianapolis, IN, USA). After 48 h, the cells were split into a selective medium containing G418 (800 µg/mL) for pIND/IGFBP-3 and zeocin (100 µg/mL) for pVgRXR, and the foci were picked to generate a stable cell line expressing pVgRXR and pIND/IGFBP-3. Ponasterone A, an ecdysone analog inducing heterodimers of RXR and VgEcR to bind the hybrid ecdysone response element in the pIND vector, was used to express IGFBP-3 in an inducible manner. MCF-7 cells transfected with pVgRXR were used as a negative control (EcR cells).

### hTERT plasmid transfections in human normal breast cells

Human normal breast MCF-12F cells were purchased from the ATCC. MCF-12F cells were cultured in DMEM:F-12 media with 20 ng/mL epidermal growth factor, 100 ng/mL cholera toxin, 0.01 mg/mL human insulin, 500 ng/mL hydrocortisone, and 5% chelex-treated horse serum. Cells were incubated at 37 °C in a humidified incubator with 5% CO_2_. Lipofectamine 2000 transfection reagent and hTERT plasmid DNA were purchased from Invitrogen and OriGene (Rockville, MD, USA), respectively. Cells were seeded in 6-well plates at a concentration of 2 × 10^5^ cells/well. At 70–80% confluence, 500 ng hTERT plasmid DNA was transfected into MCF-12F cells with antibiotic-free Opti-MEM using Lipofectamine 2000 according to the manufacturer’s instructions. After incubation for 5 h, the transfected cells were washed using PBS and replaced with fresh serum-free media with designated concentrations of the exogenous IGFBP-3. The samples for immunoblotting and real-time PCR were obtained 48 h after changing the medium.

### Small interfering RNA transfection

Small interfering RNA (siRNA) pool against human IGFBP-3 (siRNA:IGFBP-3, siGENOME SMARTpool M-004777-01-0005) and mismatch control (ON-TARGETplus siCONTROL, D-001810-01-05) were purchased from Dharmacon Research (Lafayette, CO, USA). The SMARTpool siRNA against IGFBP-3 comprises four different siRNAs against four different target sequences in the IGFBP-3, but not individual siRNA. Lipofectamine 2000 was used to transfect 50 nM siRNA:IGFBP-3 into the cells. Transfected cells were incubated with 10% FBS in growth media for 24 h.

### Cell viability assay

Cell viability was performed using the CellTiter 96 aqueous one cell proliferation assay kit (Promega, Madison, WI, USA) as per the manufacturer’s instructions. Culture media was aspirated, followed by adding 20 µL of CellTiter 96 Aqueous one solution containing 100 µL of culture medium directly to culture wells and incubating for 1 h at 37 °C. The number of viable cells was measured in a 96-well plate reader at 490 nm. We used 50 nM siRNA:IGFBP-3 to suppress the expression of IGFBP-3 or 25 µM Z-VAD-fmk to suppress the overall caspase activity, which was shown to completely inhibit apoptosis in the same cell line in our previous study^14^.

### Flow cytometric analysis of non-cycling cells

A modified BrdU–Hoechst quenching technique was performed to quantify the fraction of non-cycling cells according to a previously described method^56^. Cells were grown in 6-well plates until 60–70% confluent, and then the media were replaced with SFM in the presence or absence of 15 µM ponasterone A or 100 mM BrdU for 24 h. Both attached and floating cells were harvested via trypsinization, pelleted at 1000 rpm for 5 min, and washed thrice with PBS. Each cell sample was stored in DMEM containing 10% DMSO at − 20 °C until analyzed. Cells were thawed and stained with 2 µg/mL Hoechst 33,258 (Sigma) for 10 min at 4 °C. Cells were analyzed for cell number versus Hoechst fluorescence using an LSR-II flow cytometer (Becton Dickinson, Franklin Lakes, NJ, USA) equipped with two argon lasers. The first and the second laser were tuned to 488 nm (15 mW output) and 360 nm (10 mW output), respectively. The mean percentage of the non-cycling cells was determined. Three independent experiments were completed for this analysis.

### Senescence-associated β-galactosidase staining

Senescence-associated ß-galactosidase (SA-ß-galactosidase) staining was performed according to a previously described method^34^. Sub-confluent cells were washed twice in PBS, fixed with 2% formaldehyde/0.2% glutaraldehyde in PBS, and rinsed twice with PBS. The cells were stained with a staining solution (0.1% 5-bromo-4-chloro-3-indolyl-ß-d-galactoside, 40 mM citric acid, sodium phosphate (pH 6.0), 5 mM potassium ferrocyanide, 5 mM potassium ferricyanide, 150 mM NaCl, and 2 mM MgCl_2_). Then, the cells were incubated for 6 h at 37 °C. The cells were rinsed twice with PBS after incubation. Approximately 1000 cells in each plate were counted at × 200 magnification. The proportion of cells exhibiting a medium- to dark-blue stain, indicative of SA-ß-galactosidase activity, was then scored. Duplicate plates/cell lines were used in each experiment. Three independent experiments were completed.

### Senescence β-galactosidase activity

β-Galactosidase activity in senescence was measured using the senescence β-galactosidase activity assay kit (Cell Signaling Technology, Beverly, MA, USA) according to the manufacturer’s instructions. Cells were washed with cold 1× PBS. After removal of the wash solution, cold 1× Senescence Cell Lysis Buffer with 1.0 mM PMSF was added. After incubation on ice for 5 min, the cell lysate was centrifuged at 14,000 rpm for 5 min at 4 °C. The supernatant was transferred to a fresh tube, and the total protein concentration of each cell lysate was determined using a protein assay. Fifty microliters of the cell lysate was transferred to a 96-well plate, and 50 μL of freshly prepared 2× assay buffer (10 mM BME in 2× senescence reaction buffer) was added. The plate was incubated at 37 °C in the dark for 2 h. Fifty microliters of the reaction mixture was transferred to a 96-well black opaque plate, and 200 μL of senescence stop solution was added to each well. The 96-well plate was read with a fluorescence plate reader set with excitation at 360 nm and emission at 465 nm.

### Preparation of cell lysates

Cell lysates were prepared as described previously with minor modifications^25^. In brief, confluent cells were washed with cold PBS and then scraped from plates in the presence of cold lysis buffer containing 20 mM Tris (pH 8.0), 1% NP-40, 0.1% sodium dodecyl sulfate (SDS), 150 mM NaCl, 0.5% sodium deoxycholate, and complete protease inhibitor mixture (Boehringer Mannheim). Floating cells in the conditioned media were collected via centrifugation and resuspended in cold lysis buffer. Cell lysates obtained from confluent and floating cells were centrifuged for 15 min at 4 °C to remove cell debris. The aliquots were stored at − 70 °C until further use.

### Western blotting

The cell lysates were electrophoresed on a 12% SDS–polyacrylamide gel under reducing conditions and electro-transferred onto polyvinylidene fluoride membranes. A standard blotting protocol was performed using anti-human IGFBP-3 antibodies (1:500 dilution) followed by peroxidase-conjugated IgG (1:3,000 dilution). Human TERT protein (120 kDa) level was evaluated using the diluted antibody (1:1000 dilution) after electrophoresis on an 8% gel. Immunoreactive proteins were detected using enhanced chemiluminescence (Amersham Biosciences, Little Chalfont, UK).

### Telomeric repeat amplification protocol assay

Telomerase activity was determined by the telomeric repeat amplification protocol (TRAP) using the TeloTAGGG Telomerase PCR ELISA PLUS kit (Roche, Penzberg, Germany) in accordance with the manufacturer’s instructions. Briefly, 2 × 10^6^ cells were lysed in 200 µL of ice-cold lysis buffer for 30 min. Then, the lysate was centrifuged at 16,000×*g* for 20 min at 4 °C, and the supernatants were collected. Control templates were included in the kit, which contained positive-telomerase template DNA with the same sequence as a telomerase product with eight telomeric repeats. Two microliters of each extract (corresponding to 2 × 10^3^ cell equivalents) were assayed for telomerase activity in a 50 µL reaction using a modified TRAP assay. Telomeric repeats were added to a biotin-labeled primer during the first telomerase-mediated extension reaction. Then, the elongation products were amplified in a PTC-220 DNA Engine Dyad PCR machine (MJ Research, Waltham, MA, USA). An aliquot of the PCR product was denatured, hybridized to a DIG-labeled, telomeric repeat-specific probe, and bound to a streptavidin-coated 96-well plate. Finally, the immobilized PCR product was detected with an anti-DIG-horseradish peroxidase coupled antibody, visualized by a color reaction product using the substrate tetramethylbenzidine, and semi-quantified photometrically at 450 nm using a FLUORstar Optima microplate reader (BMG Labtechnologies, Germany). Negative controls were prepared from each cell extract by heat-inactivating the telomerase for 10 min at 85 °C.

### Reverse-transcription quantitative PCR

Total RNA was isolated from the cells using a TRIzol reagent (Invitrogen). Five micrograms of total RNA was subjected to reverse transcription using Superscript TM III first-strand synthesis system (Invitrogen) according to the manufacturer’s instructions. Real-time PCR was performed in 20 µL reaction mixtures containing cDNA, TaqMan primer pairs, and TaqMan universal PCR master mix (Applied Biosystems, Foster City, CA, USA). The primer pairs were obtained from Applied Biosystems (Hs00972650 for hTERT, TR-ANY for hTR, and Hs99999903 for β-actin). Amplification was performed in duplicate with the ABI 7300 system (Applied Biosystems) with the following profile: 50 °C for 2 min, 95 °C for 10 min, and 40 cycles of 95 °C for 15 s and 60 °C for 1 min. Gene expression for each sample was expressed as the threshold cycle (Ct) normalized to β-actin (ΔCt). The ΔCt values were compared between samples from ponasterone A-treated cells and control samples (cells in serum-free media) to calculate ΔΔCt. The final comparison of transcript ratios between samples was given as 2^ΔΔCt^^30^.

### Densitometric measurement

Densitometric measurements of immunoblots were performed using a Bio-Rad GS-670 Imaging densitometer (Bio-Rad, Melville, NY, USA).

### Statistical analysis

Quantitative data are presented as the mean ± standard deviation. For multiple comparisons, the data were analyzed with one-way ANOVA and Bonferroni post-hoc analysis. The statistical significance was defined as a p-value < 0.05. All analyses were performed using SPSS software version 26.0 for Windows (IBM, Chicago, IL, USA).

## Supplementary Information

Supplementary Figure S1.

Supplementary Figure S2.

Supplementary Table S1.

## Data Availability

The data presented in this study are available on request from the corresponding author.
