# Supplementary figures and images for: Insulin-like growth factor binding protein-3 induces senescence by inhibiting telomerase activity in MCF-7 breast cancer cells
Source: Sci Rep. 2023 May 30;13:8739. doi: 10.1038/s41598-023-35291-5 (PMC10229562; doi:10.1038/s41598-023-35291-5)

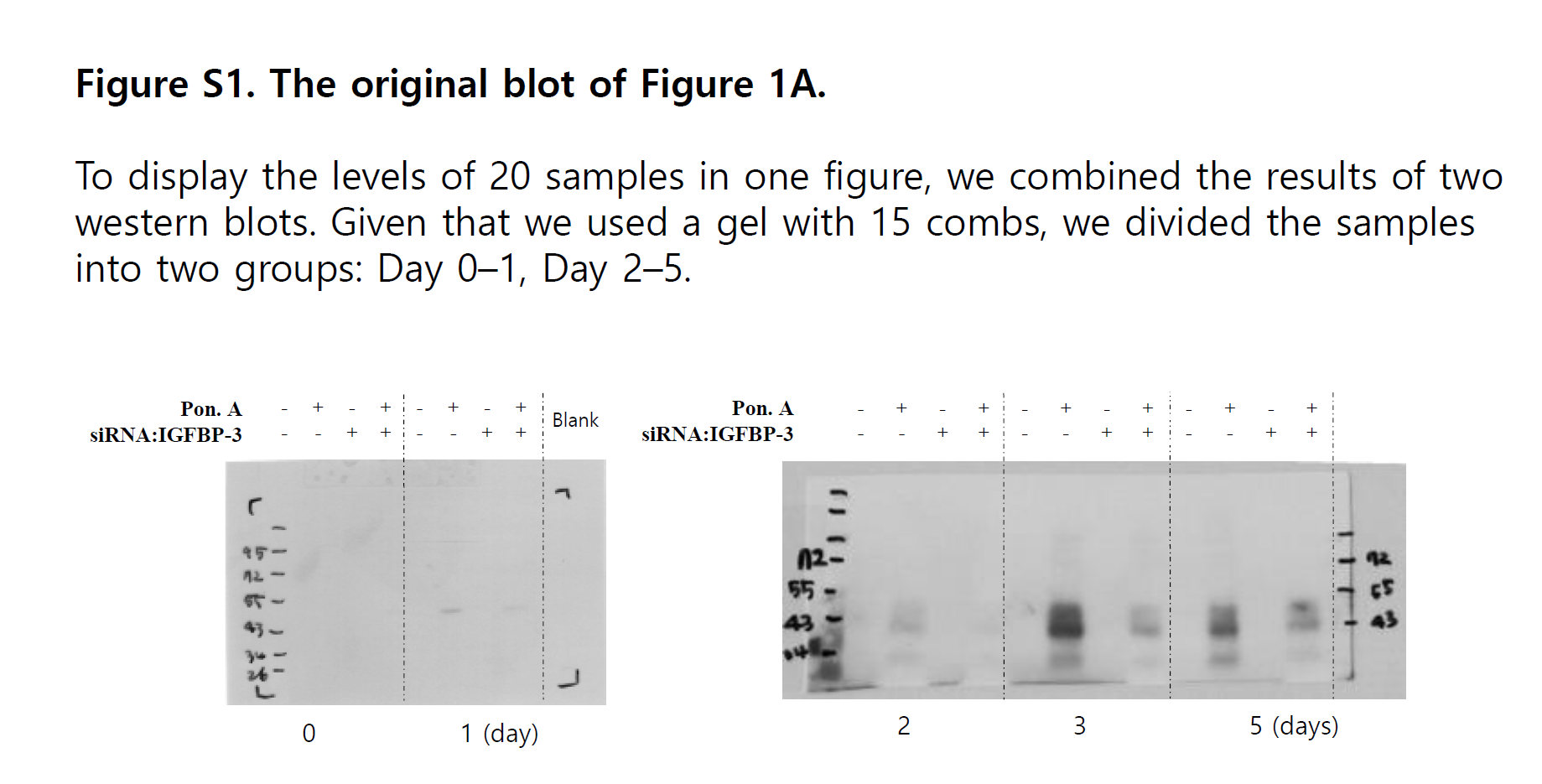

Supplement: Supplementary file 1 — Supplementary Figure S1. [file 41598_2023_35291_MOESM1_ESM.tif]

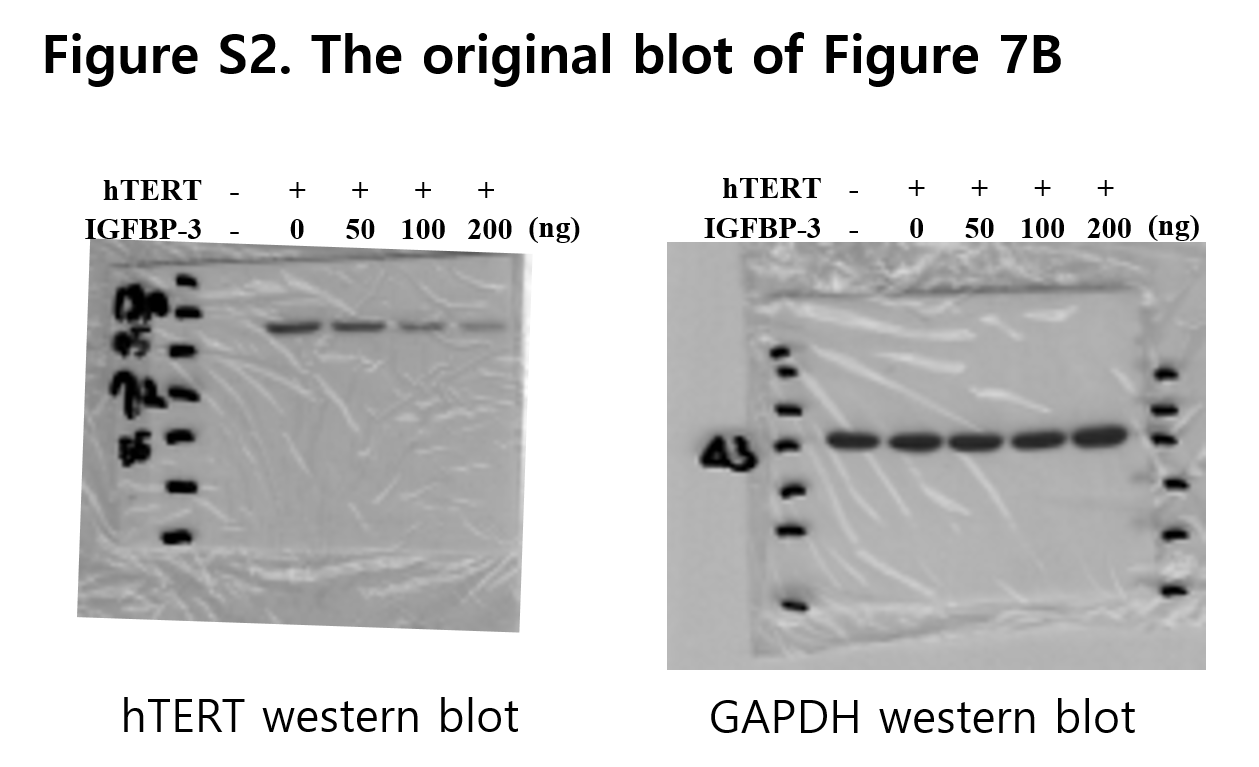

Supplement: Supplementary file 2 — Supplementary Figure S2. [file 41598_2023_35291_MOESM2_ESM.tif]
